# Tetanus in Injecting Drug Users, United Kingdom

**DOI:** 10.3201/eid1204.050599

**Published:** 2006-04

**Authors:** Susan J.M. Hahné, Joanne M. White, Natasha S. Crowcroft, Moira M. Brett, Robert C. George, Nick J. Beeching, Kirsty Roy, David Goldberg

**Affiliations:** * Health Protection Agency, London, United Kingdom;; † Royal Liverpool University Hospital, Liverpool, United Kingdom;; ‡ Scottish Centre for Infection and Environmental Health, Glasgow, United Kingdom

**Keywords:** Tetanus, heroin, street drugs, substance abuse, intravenous, immunization, Clostridium tetani, letter

**To the Editor:** The epidemiology of tetanus in the United Kingdom changed in 2003 when a cluster of cases in injecting drug users (IDUs) occurred (*1**,**2*). Before 2003, the incidence of tetanus was low in the United Kingdom, with occasional cases predominantly in unvaccinated elderly persons (*3*). The situation contrasted with the United States where injecting drug use is commonly reported among persons with tetanus (*4*).

We investigated the UK cluster to identify the source of infection and opportunities for prevention. We ascertained cases through statutory and nonstatutory reporting to the Health Protection Agency and collected additional information on IDUs for all reported cases of tetanus since January 1, 2003, by adapting the existing enhanced tetanus surveillance. A case was defined as mild-to-moderate trismus and at least 1 of the following: spasticity, dysphagia, respiratory embarrassment, spasms, autonomic dysfunction, in a person who injected drugs in the month before symptom onset.

Twenty-five cases were reported from July 2003 to September 2004 (Figure). Thirteen (50%) were women; the median age of male and female patients was 39 and 32 years of age, respectively (range 20–53, p = 0.1). Twenty patients were white, and 1 was Chinese (information was missing for 4). None reported travel overseas before becoming sick. Seventeen of 21 patients with information reported having injected heroin intramuscularly or subcutaneously (popping) or having missed veins. Most patients (16/25) came to the hospital with severe generalized tetanus. Injection site infections were common (17/19).

**Figure:**
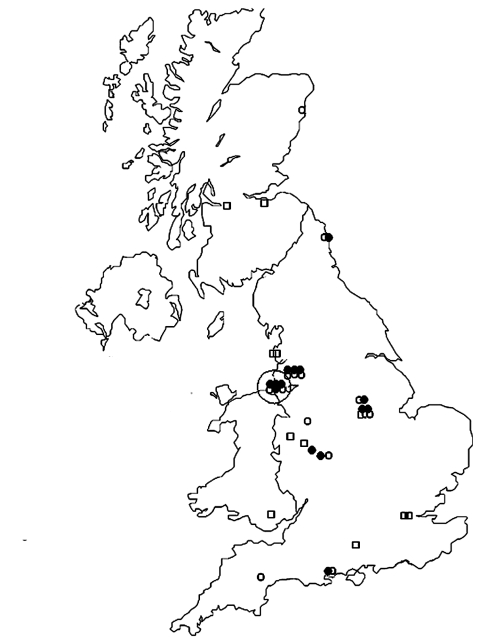
Cases of tetanus in injecting drug users by residence (25 cases) and place from which heroin was supplied (14 cases with information), United Kingdom, July 2003–September 2004. The large circle indicates the Liverpool area. Squares indicate the residence of patients for whom the origin of heroin was not reported, open circles indicate the residence of patients for whom the origin of heroin was reported, and solid circles indicate the origin of heroin.

Two patients died (case fatality 8%). Of 23 survivors, 2 had mild disease and 21 required intensive treatment for a median of 40 days (range 24–65 in 15 cases with complete information). Tetanus immunization status available for 20 case-patients (based on medical records or patient and parental recall) indicated that only 1 patient (with severe disease) had received the 5 doses necessary for complete coverage. Nine patients were never vaccinated. Twelve of 14 patients tested for tetanus immunity on admission by a standard indirect enzyme-linked immunosorbent assay had antibody levels lower than the cutoff value for protection (<0.1 IU/mL). One patient with severe disease had a level just above the cutoff value and 1 patient with mild disease had a protective antibody level. *Clostridium tetani* was isolated from 2 patients; tetanus toxin was detected in serum from 1 and also from another patient. Other anaerobes, including *C*. *novyi*, *C*. *histolyticum*, and *C*. *perfringens*, were isolated from injection site wounds of 3 patients. A heroin sample from 1 patient was tested by polymerase chain reaction, but no evidence of tetanus contamination was found (J. McLauchlin, pers. comm.). One (fatal) case of tetanus was reported from the Netherlands through the European Monitoring Centre for Drugs and Drug Addiction (L. Wiessing, pers. comm.).

Tetanus, arguably the oldest infection associated with IDU (*5*), can be caused by spore contamination during production, distribution, storage, cutting, reconstitution, and injection of drugs. The widespread distribution and temporal clustering (*1*) of cases in the United Kingdom suggest that its cause was contamination of heroin rather than changes in injecting practices. This finding is consistent with results of a similar investigation of a cluster of *C*. *botulinum* in IDUs in California (*6*). Only 1 case was reported outside the United Kingdom, which suggests that contamination occurred within the United Kingdom. The pronounced clustering of the place from which the heroin was supplied, compared with the residence of IDUs (Figure), is consistent with contaminated heroin having been distributed from Liverpool.

Intramuscular or subcutaneous injection of heroin was common among case-patients. This was also found in a large international outbreak of *C*. *novyi* in IDUs (*7*) and a botulism outbreak in IDUs in California (*6*), and is consistent with the obligate anaerobe characteristic of *Clostridium* spp. In our cluster and in other outbreaks, women and older injectors were overrepresented compared with demographic estimates of IDUs (*8*). Women and long-term IDUs may have difficulty accessing veins and frequently inject intramuscularly or subcutaneously. Furthermore, tetanus immunity is more likely to be inadequate or have waned with age.

The reasons for emergence of *Clostridium* infections in IDUs in the United Kingdom remain speculative (*9*). They include an increase in contamination of heroin and an aging cohort of heroin users who are more likely to use popping as the mode of injection.

In the United Kingdom, 5 doses of tetanus toxoid–containing vaccine at appropriate intervals are considered to provide lifelong protection, as long as tetanus-prone wounds are treated with tetanus immunoglobulin (*10*). Only 1 case in the present cluster received the recommended 5 doses of tetanus vaccine. This coverage is lower than what might be expected. During the period in which most of the patients were born (1964–1984), primary immunization coverage increased from 75% to 85% (http://www.hpa.org.uk). Since IDUs are at risk for *Clostridium* infections (*9*), drug action teams, needle exchange programs, prison staff, and clinicians should ensure that IDUs are vaccinated against tetanus and educated about signs and symptoms of soft tissue infections that require prompt medical intervention.
